# Drs. Cone and Mott’s Case of Monstrosity

**Published:** 1852-01

**Authors:** E. D. Cone

**Affiliations:** Hillsdale, Mich.


ART. III.—*Drs. Cone and Mott's Case of Monstrosity.* Reported for the Buffalo Medical Journal, by E. D. Cone, M. D., Hillsdale, Mich.

On the 30th day of September last, my partner, James N. Mott, M. D., was summoned to attend Mrs. N——, of the town of Adams, (this county,) then in confinement with her first child. On arriving, we saw that her pains were regular, the "bag of waters" having ruptured, and that she was in the second stage of labor. In making an examination, it was with some difficulty that we ascertained that the head presented, owing to the absence of the *Fontanels*, and the presentation of a soft and yielding tumor. In consequence of the knowledge elicited by the examination, we were expecting to have a case of monstrosity, and informed the husband and attendants of our expectations, thereby preparing them for the coming event, that they might act accordingly, as we deemed it prudent that the mother should not be embarrassed in her then condition by beholding it, nor excited by any imprudent or inconsiderate expressions. After a somewhat protracted labor, at the full period of gestation, the woman was delivered of an *acrania* male fœtus, weighing nine and one-fourth pounds, of remarkable external appearance. Its body and extremities are well formed and natural, except a curvature

&c. of the spine (to be described presently;) it has no neck; the cervical vertebræ are (apparently) wanting, as the head rests directly on the shoulders, and the chin upon the sternum. Its face is normal, except that its eyes are very prominent, there being no forehead. The *frontal*, the two *parietal*, the *squamous portion* of the *temporal*, as also the most of the *occipital* bones are wanting; likewise the contents of the cranium down to its base; the deficient portion is covered with the common integuments and hair. There is a prominence over the eyes caused by the optic vessels, which, together with the tops of its ears, are higher than the head; there is situated directly over the superior portion of the medulla oblongata a vascular tumor, divided distinctly into two hemispheres, oval and convoluted; it has a veinous appearance in consequence of numerous tortuous veins passing over it, and the same can be felt in its substance. The tumor is slightly elevated above the base of the cranium; it measures in width about two and one-half inches, and four inches long, and hangs down upon the dorsal vertebræ, which are devoid of skin or other covering except a thin membrane on which the tumor rests. The spinous processes of the dorsal vertebræ are wanting, and they appear to be flattened; they are much broader, laterally, than natural; they also form quite a posterior curvature.

I have thus described quite minutely the external appearance; the internal structure we are ignorant of, as no examination was allowed; but we finally succeeded, by earnest and repeated solicitations, in obtaining the specimen for preservation, which can be seen at our office.

The mother is a robust, well developed, and usually healthy American lady, aged 22; during the second or third month of gestation, she recollects witnessing a dog seize a cat by the nape of the neck and shake it violently. I may remark that the fœtus was still-born, but evidently had lived until quite recently, and I am inclined to think that it would have been alive, had not the mother met with an accident by falling, about a week previous to her confinement. The "after-birth" was delivered within the usual time, and after ordinary convalescence, she fully recovered.

Dr. Purple's case, published in the N. Y. Jour. of Med. &c.. Vol. 5, No. 1, July, 1850, is somewhat analogous, but inasmuch as this case differs from his in many respects, I presume it would be of sufficient interest to your numerous readers to warrant my reporting it.

On the 8th of September last, I was called to attend a Mrs. D——, in confinement with her first child. After ordinary labor, at full period of gestation, she was delivered of a female child weighing 10 lbs. 2 oz., perfectly formed and natural, except *talipes valgus* of *right foot.* The mother, during gestation, fell, turning the *right foot out*, (her ankle having been weak for some time previous, owing to a sprain,) in the same manner as the child was found to be at birth.

*Query.*—Do the above detailed circumstances fully account for the monstrosity and deformity? Do external impressions of the mother, received during gestation, affect the development of the fœtus in utero? If not, how are the above cases, together with many others of a similar nature, to be accounted for?

